# Healthcare service user-reported quality of care in Malawi: a national multifacility cross-sectional study

**DOI:** 10.1136/bmjopen-2025-115140

**Published:** 2026-06-30

**Authors:** Joseph H Collins, Wiktoria Tafesse, Precious Chitsulo, Timothy B Hallett, Eva Janoušková, Joseph Mfutso-Bengo, Emmanuel Mnjowe, Sakshi Mohan, Watipaso Mulwafu, Victor Mwapasa, Dominic Nkhoma, Bingling She, Mariana Suarez, Tim Colbourn

**Affiliations:** 1 Global Business School for Health, University College London, London, UK; 2 Centre for Health Economics, University of York, York, UK; 3 Health Economics and Policy Unit, Kamuzu University of Health Sciences, Lilongwe, Malawi; 4 MRC Centre for Global Infectious Disease Analysis, Imperial College London, London, UK; 5 Institute for Global Health, University College London, London, UK; 6 Health Systems and Capacity Development, East Central and Southern Africa Health Community, Arusha, Tanzania; 7 Department of Epidemiology and Biostatistics, Kamuzu University of Health Sciences, Blantrye, Malawi

**Keywords:** Health Services, International health services, Quality in health care

## Abstract

**Abstract:**

**Objective:**

Improving healthcare service user-reported quality of care is one of the three core objectives of the Malawian government’s health sector strategic plan. As such, a robust understanding of service user-reported quality is crucial to inform the development of patient-centred services. This study aimed to explore how service users in Malawi evaluate the quality of the healthcare they receive across services and facilities and to investigate the association between individual and health service characteristics and reported quality of care.

**Design:**

A national multifacility cross-sectional study using service user exit interview data in which all central hospitals were sampled and remaining facilities were selected via random stratified sampling. Participants were recruited via random selection based on daily patient load within sampled facilities. Descriptive statistics of service user-reported quality of care were estimated in addition to multinomial logistic regression analyses used to determine the association between patient and healthcare characteristics and perceived care quality.

**Setting(s):**

30 health facilities across 15 districts in Malawi.

**Participants:**

4181 respondents surveyed after completing their visit and exiting healthcare facilities between January and May 2024.

**Primary outcome measures:**

Overall service user-reported quality of care for the facility visit as a categorical variable. This was derived from the Likert scale survey statement assessing ‘overall’ quality of the participant’s visit, with three responses: ‘very good’, ‘good’, ‘neutral–very bad’.

**Results:**

Quality of care was reported as being high with 58% of respondents rating care as ‘good’ and 35% as ‘very good’, with some variation by ‘dimension’ of care (eg, treatment availability). Positive or negative perceptions of care quality were associated with age, sex, education level, illness severity, previously seeking care, referral, facility type, non-governmental facility ownership, service area, access to medication and payment of fees.

**Conclusions:**

Most healthcare service users in Malawi report receiving high-quality care, although a minority report the quality to be inadequate. Action to address gaps in service delivery, such as improving the availability of required medicines, could address the poor perceptions of quality held by the minority of interviewed service users.

**STRENGTHS AND LIMITATIONS OF THIS STUDY**

- The facility sample was explicitly stratified by facility type, ownership and patient volume with random selection within each stratum enhancing coverage across these dimensions.
- A large sample of service users who had accessed care across a broad variety of clinical settings were recruited from included facilities increasing confidence that findings reflect experiences across the health system.
- As this study was cross-sectional, causal relationships cannot be confirmed.
- Service users who opted to participate may have some important differences compared with the wider population (eg, those who did not seek care, those who died during treatment).

## Introduction

 Despite the concerted efforts of national governments during the Sustainable Development Goal era, including those prompted by global initiatives such as the Lancet Global Health Commission on High Quality Health Systems^1^ and WHO coordinated Quality of Care Network,^2^ there remains substantial international and intranational variation in the quality of healthcare.^3 4^ Poor quality health services are a key barrier to progress in global public health, given the relationship between quality and health outcomes.^5^ This is particularly true in low- and middle-income countries (LMICs) such as Malawi, in which system-wide drivers of poor-quality care include limited availability of medical consumables, healthcare workers (HCWs) and functional infrastructure,^6^ driven by significant constraints on resources for health,^7^ compounded by a growing burden of communicable and non-communicable disease.^8^

While understanding the system-level drivers of poor quality healthcare is imperative to guide quality improvement, empirical evaluation of how populations in LMICs perceive quality of care is a rapidly developing area of academic and policy interest.^4 9^ The concepts most commonly discussed in relation to ‘perceived quality’ include ‘patient experience’, relating to service users’ perceptions of the processes of care,^10^ and patient satisfaction, relating to whether the care received has met their expectations. Additionally, recent large scale evaluations within LMICs have sought to evaluate perceptions of care quality directly, by asking service users to explicitly rate the quality of their interaction with the health system on an ordinal scale.^4 9^ For clarity, given that direct service-user evaluations of quality^4 9^ are likely to be influenced by expectations, we refer to these measures, including patient satisfaction, collectively as service user-reported quality throughout this paper. Although outcomes from such measures do not necessarily correlate with outcomes from objective measures, in which observed care is compared with predefined quality or clinical indicators,^11^ they provide highly important and policy-relevant evidence. Beyond being effective and safe, high-quality healthcare is people-centred and responsive to the preferences, needs and values of service users,^1^ who, as the consumers of healthcare, are best placed to assess the quality of a product.^12^ Vitally, service user-reported quality is a driver of key behaviours related to healthcare utilisation^13–15^ and addresses gaps in objective measures by capturing acceptability and dimensions of interpersonal quality (eg, dignity, respect).

Malawi’s Ministry of Health (MoH) continues to demonstrate a clear commitment to the universal delivery of high-quality and people-centred health services through the development of the Quality Management Directorate in 2016 and operationalisation of the most recent Health Sector Strategic Plan III (2023–2030).^16^ Key quality improvement initiatives include the implementation of the first national Quality Management Policy (2016–2022)^17^ and a novel quality rating programme that standardises facility assessments and designates ‘Centres of Excellence’.^16^ High-quality health systems must not only be reliable and adaptive,^18^ but they must also be trusted by users and responsive to their needs.^19^ Thus, the MoH has placed improving service user-reported quality as central in moving towards Universal Health Coverage.^16^

However, it is difficult to draw conclusions related to how service users perceive the quality of the services they receive in Malawi from the existing empirical literature given the heterogeneity of included outcome definitions, samples and health services. Most studies have explored service user-reported quality in relation to one or two services or service areas^6 20–28^ with a particular focus on reproductive and maternal health.^6 20 21 25–28^ Where quality of multiple services is explored, this is largely done within single facilities, which are mostly at the tertiary level of the health system.^21 29^ Studies focus almost exclusively on adolescent or adult patients,^20–28^ with few studies exploring service user-reported quality of care for children.^6^

Therefore, this study seeks to (1) explore how service users in Malawi perceive the quality of the healthcare they receive across a variety of healthcare services and facilities and (2) investigate the association between key individual and health service characteristics and reported quality of care.

## Methods

### Study design

We report here an analysis of data collected as part of the Thanzi La Mawa (TLM) study—a cross-sectional and mixed methods study intended to improve understanding of healthcare delivery in Malawi and to inform Malawi’s first whole-health system mathematical model (the Thanzi La Onse model).^30^ The TLM study included facility audits, time-and-motion data collection, service user exit and follow-up surveys, and qualitative interviews of facility managers. The TLM study design and data-collection processes have been previously described.^31^ Data analysed for this study was collected primarily through service user exit-surveys and we have used the Strengthening the Reporting of Observational Studies in Epidemiology (STROBE) guidelines on cross-sectional studies^32^ to report these results (online supplemental file 1, table S1).

### Setting

The service user exit-survey component of the TLM study was conducted at 30 health facilities across 15 districts in Malawi, namely Blantyre, Chiradzulu, Dedza, Karonga, Likoma, Lilongwe, Mzimba, Mzuzu, Neno, Nkhata Bay, Nsanje, Ntcheu, Rumphi, Thyolo and Zomba. Healthcare services are delivered in Malawi via a three-level system consisting of primary, secondary and tertiary levels, with a referral system operating between them.^16^ At the primary level, services are delivered to the community via health posts, dispensaries, health centres and community hospitals. At the secondary level, inpatient and outpatient services are delivered via government district hospitals and other non-state hospitals of similar capacity. Tertiary level services are provided by four central hospitals and one mental hospital, which act as referral centres for secondary hospitals. While the health system consists of a mix of public, private-for-profit and private not-for-profit service providers, the public sector, which delivers services for free at the point of use, provides the majority of healthcare.^6^ The private sector provides the remainder of services, with the Christian Health Association of Malawi (CHAM), a faith-based private-not-for-profit organisation, being the largest private provider.^6^ The government has a Memorandum of Understanding with CHAM, under which neither party constructs a facility within a 7 km radius of the other. In turn, Service Level Agreements between the Government and CHAM providers reimburse CHAM for part of the service costs, thereby enabling users to access contracted services free of charge.

### Survey design, participants and data collection

The exit-survey was developed for the conduct of this study. Face and content validation of the initial survey design was conducted by the research team. Prior to final data collection, during January 2024, the survey tool was piloted following initial review of the instruments with enumerators and the research team. The survey tool was initially piloted at Kabudula Community Hospital in Lilongwe, after which it was reviewed and refined, followed by a second pilot at Area 25 Health Centre in Lilongwe. Study data were collected between January and May 2024 at 30 health facilities including all four central (tertiary) hospitals (Queen Elizabeth, Zomba, Kamuzu, Mzuzu) and Zomba Mental Hospital. To capture variation across relevant facility contexts in Malawi 25 facilities were selected via stratified random sampling. Facilities were selected using a random number generator in Microsoft Excel from the National Master Health Facility Register following stratification by facility type (health centre, community hospital, secondary hospital), patient volume (relatively lower or higher), ownership (MOH or CHAM) and location (urban or rural). The sampling frame was not powered for national representativeness and as such we have not used sampling weights in our analysis. A full list of included facilities is available in online supplemental file 1, table S2.

Exit-survey data were collected by five teams of enumerators across enrolled facilities using KoboToolbox,^33^ a secure data-collection software allowing for offline data capture and data validation, following enumerator training and initial data-collection tool piloting. Enumerators randomly selected potential participants from adults exiting the facility after being discharged or accompanying someone who was discharged. To survey a random sample of patients, participant selection was based on daily patient load (estimated via facility registers or waiting room attendance). For example, if enumerators aimed to survey 15 patients per day, every Xth patient was interviewed to reach this number of surveys. Practically, given that there was one enumerator per facility, a new participant was approached every 15–20 min, which is the average time it took to complete the survey. The survey was exclusively administered by enumerators. The survey was formulated and coded in English and translated into Chichewa prior to data collection. Rarely, the survey was translated by enumerators to Tumbuka if required during data collection. Pretranslation of key terms was conducted prior to data collection during the piloting phase to reduce the risk of measurement error. Informed consent was obtained prior to participation. Enumerators were trained to provide all participants with clear information about independence between the research team and facility management and to reassure them that participation would have no consequences relating to future treatment. Additionally, enumerators were trained to conduct surveys completely out of earshot of all facility workers. There were no restrictions on eligibility for participation.

The service user exit-survey, available in online supplemental file 2, consisted of possible 68 questions across seven areas: patient details (11 questions), facility visit (14 questions), patients’ access to medicines (2 questions), patients’ persistence in care-seeking (3 questions), costs and opportunity costs of attending facility (11 questions), household assets (17 questions) and perceptions of quality of care (10 questions). In total, data were collected from 4181 respondents across all included facilities.

Following data collection, data were extracted from KoboToolbox to Microsoft Excel (Office 365, V.16.94), anonymised and imported into Stata/SE V.18.0 (Statacorp) for data cleaning and analysis. Online supplemental file 3 contains the Stata .do file describing the data cleaning procedures.

### Patient and public involvement

Patients or the public were not involved in the design or conduct of this research. Dissemination of our findings will occur at a research meeting held at Kamuzu University of Health Sciences which is attended by representatives of civil society groups.

### Statistical analysis

Characteristics of the sample, the included facilities and the service user-reported quality of care have been described first with descriptive statistics. The association between variables and the primary outcome, overall service user-reported quality of care for the facility visit, was explored via multinomial logistic regression. The primary outcome of this analysis was a categorical variable derived from the Likert scale survey statement assessing ‘overall’ quality of the participants visit, with three responses: ‘very good’, ‘good’, ‘neutral–very bad’. A multinomial analysis using this outcome variable, as opposed to ordered logistic regression using the Likert scale survey statement, was necessary for several reasons. First, this approach accounts for the small number of respondents who rated overall quality as ‘bad’ or ‘very bad’. Second, a core assumption of an ordered model is proportional odds, in which covariates are assumed to have a constant effect across all ‘thresholds’ for the ordinal outcome.^34^ We evaluated this formally, first by fitting an ordered logistic regression model and conducting the Brant test.^34^ However, this test failed to converge, likely due to the sparse responses in key outcome categories and we did not interpret these tests for this outcome. For completeness, we evaluated whether using an ordered model would be appropriate with the derived outcome variable, but the Brant test indicated that the proportional odds assumption was violated (p=0.00).

Covariate selection was informed by the existing literature describing determinants of service user-reported quality of care or satisfaction reported in studies from Malawi^6 20–23 25–28^ and a systematic review evaluating determinants of service user-reported satisfaction with care delivered across a given health system.^35^ All covariates included are described in table 1. Where covariates are categorical or binary variables derived from survey questions, online supplemental file 1, table S3 describes the relevant derivation.

**Table 1:** Description of covariates

Covariate	Description
Age	Service users’ age in 10-year units (age in years/10)
Sex	Service users’ sex (male/female)
Education level*	Respondent’s education level (none/some primary education, primary/some secondary education, secondary and above)
Wealth quintile*	Respondent’s wealth quintile (1–5) calculated via Principal Component Analysis using respondent’s assets
Illness severity	Self-reported severity of illness for service user (healthy, mild symptoms, moderate symptoms, severe/life-threatening symptoms)
Reason for attendance*	Respondent’s reason for attending facility (adult seeking care, adult accompanying a child, adult accompanying an adult)
Facility type	Type of facility (health centre, community hospital, district hospital, tertiary hospital)
Facility ownership	Ownership of visited facility (governmental, non-governmental)
Facility location	Location of visited facility (rural/urban)
Travel time to facility	Time taken to reach visited facility for care in 10-minute units (travel time in minutes/10)
Service area	Service area attended during visit to facility (disease clinics, emergency department, maternity or gynaecology inpatients, maternity or gynaecology outpatients, other outpatients, other inpatients, surgery)
Time spent with healthcare worker (HCW)	Self-reported total time spent with any HCWs during visit to facility in 10-minute units (self-reported time/10)
Any fees paid to the facility	Whether any fees were paid directly to the health facility during this visit (yes, no)
Any inpatient days	Whether the service user spent any days at the facility as an inpatient (yes, no)
Accessed required medication	Whether medication that was prescribed could be accessed (yes, no, none prescribed)
Referral	Whether the service user was referred from another facility (yes, no)
Previously sought care	Whether the service user had sought care before for this condition (yes, no)

* These covariates relate to the survey respondents who may either be the service user or the person accompanying them.

We developed three multivariable models, each incrementally incorporating additional groups of variables. Model 1 included variables relating to individual service user sociodemographic and illness characteristics (age, sex, education level, wealth quintile, illness severity, previously sought care, referral). Model 2 included all variables in model 1 plus facility characteristics (type, ownership, location) and service characteristics (service area, any inpatient days). Finally, in model 3, we add policy-relevant variables closely related to service delivery (travel time to facility, time spent with a HCW, access to required medication, any fee paid to facility). This analysis was not designed to estimate independent causal effects of a single exposure on the primary outcome; instead, we use an incremental adjustment approach to explore how associations change as groups of covariates are introduced within the model. Collinearity between covariates was assessed via variance influence factors. Model SEs were estimated using the variance-covariance estimator to account for intragroup correlation within facilities. For all tests, a p value lower than 0.05 (95% CI not crossing 1, no effect) was considered to indicate statistical significance.

Missing data were observed in six variables, including service user-reported quality of care (1.6%), and ranged from 0.4% to 10.2% of total entries (tables 2 and 3). Of the 4181 respondents, 3594 (86%) had complete data for all covariates. Individuals with missing outcome or covariate data were excluded from regression models in the primary analysis. We explored the predictors of complete data using logistic regression and identified that facility location was significantly associated with complete case status (adjusted OR 1.85, p=0.026) when accounting for clustering by facilities. This suggests some structural variation in completeness across facilities. Therefore we have conducted sensitivity analysis using multiple imputation by chained equations in which variable-specific imputation models matched to the distribution of each variable with incomplete data (multinomial logistic regression for categorical variables, logistic regression for binary variables, and linear regression for continuous variables). 30 imputed data sets were generated.

**Table 2:** Sample and facility characteristics

	Total sample
(N)	4181
Age (years)	
Mean, SD	25.7 (18.5)
Median, range	24 (0–98)
Sex	
Male	1584 (37.9%)
Female	2597 (62.1%)
Education level	
None/some primary	1750 (41.9%)
Primary/some secondary	1635 (39.1%)
Secondary and above	778 (18.6%)
Missing	18 (0.4%)
Wealth quintile	
1st (lowest)	1051 (25.1%)
2nd	654 (15.6%)
3rd	821 (19.6%)
4th	823 (19.7%)
5th	832 (19.9%)
Illness severity	
Healthy	859 (20.5%)
Mild symptoms	1667 (39.9%)
Moderate symptoms	1385 (33.1%)
Severe/life-threatening symptoms	270 (6.5%)
Reason for attendance	
Adult seeking care	2527 (60.4%)
Adult accompanying another adult	272 (6.5%)
Adult accompanying a child	1382 (33.1%)
Previously sought care	
Yes	1888 (45.2%)
No	2273 (54.4%)
Missing	20 (0.5%)
Referral	
Yes	652 (15.6%)
No	3515 (84.0%)
Missing	16 (0.4%)
Facility type	
Health centre	1149 (27.5%)
Community hospital	1330 (31.8%)
District hospital	461 (11.0%)
Tertiary hospital	1241 (29.7%)
Facility ownership	
Governmental	2667 (63.8%)
Non-governmental	1514 (36.2%)
Facility location	
Urban	2888 (69.1%)
Rural	1293 (30.9%)
Service area	
Disease clinics	514 (12.3%)
Emergency department	59 (1.4%)
Maternity/gynaecology inpatient	225 (5.4%)
Maternity/gynaecology outpatient	398 (9.5%)
Other clinics/outpatients	2696 (64.5%)
Other inpatient wards	237 (5.7%)
Surgery	52 (1.2%)
Any inpatient days	
Yes	984 (23.5%)
No	3197 (76.5%)
Travel time to facility (minutes)	
Mean, SD	65.8 (49.5)
Median, range	60 (0–450)
Missing (n, %)	61 (1.46%)
Time spent with HCW (minutes)	
Mean, SD	23.1 (40.7)
Median, range	12 (0–765)
Missing (n, %)	428 (10.2%)
Access to required medication	
Yes	3528 (84.4%)
No	348 (8.3%)
None prescribed	305 (7.3%)
Any fees paid to the facility	
Yes	859 (20.6%)
No	3322 (79.5%)

HCW, healthcare worker; SD, Standard Deviation.

**Table 3:** Service user-reported quality of care in Malawi

	Total sample (n=4181)
Health worker consultation	
Very good	1651 (39.49%)
Good	2380 (56.92%)
Neither good nor bad	71 (1.70%)
Bad	29 (0.69%)
Very bad	6 (0.14%)
Missing	44 (1.05%)
Treated with respect	
Very good	1727 (41.31%)
Good	2300 (55.01%)
Neither good nor bad	88 (2.10%)
Bad	18 (0.43%)
Very bad	6 (0.14%)
Missing	42 (1.00%)
Waiting time	
Very good	1282 (30.66%)
Good	2180 (52.14%)
Neither good nor bad	248 (5.93%)
Bad	313 (7.49%)
Very bad	104 (2.49%)
Missing	54 (1.29%)
Treatment available	
Very good	1646 (40.12%)
Good	1982 (48.31%)
Neither good nor bad	297 (7.24%)
Bad	148 (3.61%)
Very bad	30 (0.73%)
Missing	78 (1.87%)
Overall	
Very good	1450 (34.68%)
Good	2406 (57.55%)
Neither good nor bad	196 (4.69%)
Bad	52 (1.24%)
Very bad	11 (0.26%)
Missing	66 (1.58%)
Appropriate care received	
Yes	3867 (92.49%)
No	314 (7.51%)

Finally, we conducted the same multinomial analyses with outcome variables derived from each of the Likert survey questions assessing perceptions of quality relating to HCW consultation, respect, waiting time and treatment availability to provide a more comprehensive view of quality perception. The Stata .do analysis file is available as online supplemental file 4. A public version of the study data, which has been cleaned and processed so that no individual respondent can be identified, is available.^36^

## Results

### Sample characteristics

The sociodemographic and illness characteristics of the sample are summarised in table 2, alongside key characteristics of the facilities at which they were interviewed. The mean age of service users in the total sample was 25.7 years (SD 18.5). More of the service users were women (62.1%, n=2597) with nearly half of respondents having received less than a complete primary education (41.9%, n=1750) and the highest percentage of respondents were in the lowest wealth quintile (25.1%, n=1051). Respondents were most likely to report severity of illness as mild (39.9%, n=1667) which was reflected in the services they accessed with the largest proportion attending ‘other clinics/outpatients’ care (64.5%, n=2696). Respondents were evenly split between being interviewed leaving a community hospital (31.8%, n=1330), tertiary hospital (29.7%, n=1241) or health centre (27.5%, n=1149) with fewer interviewed leaving a district hospital (table 2). 63.8% (n=2667) of the service user sample received care in government facilities and 69.1% (n=2888) in urban settings.

### Service user-reported quality of care

Service user-reported quality of care is summarised in table 3. Most respondents rated the overall quality of the care they received as either ‘very good’ (34.7%, n=1450) or ‘good’ (57.6%, n=2406). Over 92% of participants responded that they felt the care they received was appropriate. When considering quality across specific dimensions there was greater variation. The greatest number of participants reported care was ‘bad’ or ‘very bad’ when asked to rate quality in relation to waiting times (‘very bad’ 2.5% (n=104), ‘bad’ 7.5% (n=313)), followed by treatment availability (‘very bad’— 0.7% (n=30), ‘bad’ 3.6% (n=148). When combining ‘neither good nor bad’, ‘bad’ and ‘very bad’ responses relating to overall quality of care for the primary outcome of the regression analysis, 6.2% (n=259) of the sample reported the overall quality of care was at this level.

### Bivariate regression analysis

Table 4 reports the independent association between the variables and the primary outcome. Where variables are categorical, we provide the level associated with the primary outcome (compared with the reference category) in parentheses. Age, education level, illness severity, reason for attendance, facility type, facility ownership, facility location, service area, any fees paid to the facility, any inpatient days, access to required medications, referral, previously seeking care were all associated with respondents having either a more negative or very positive perception of quality of care compared with perceiving care as ‘good’. Wealth quintile and time spent with HCW were only associated with respondents having a more positive perception of quality of care while travel time to facility was only associated with a more negative perception of quality of care.

**Table 4:** Bivariate analysis of individual and health service factors and service user-reported quality of care

	Service user-reported quality of care unadjusted relative risk ratio (95% CI) (reference outcome–good)		
Variables	Neutral–very bad	Very good	Observations
Age (10-year units)	1.13*** (1.06 to 1.21)	1.04** (1.00 to 1.08)	4115
Sex (Ref–female)			
Male	1.14 (0.88 to 1.48)	0.90 (0.79 to 1.03)	4115
Education level (Ref–none to some primary)			4098
Primary to some secondary	0.66*** (0.50 to 0.88)	1.17** (1.01 to 1.36)	
Secondary and above	0.76 (0.52 to 1.11)	1.90*** (1.59. 2.27)	
Wealth quintile (Ref–first quintile (lowest)			4155
Second quintile	1.00 (0.67 to 1.48)	1.11 (0.89 to 1.37)	
Third quintile	0.91 (0.62 to 1.33)	1.17 (0.96 to 1.43)	
Fourth quintile	0.98 (0.67 to 1.43)	1.35*** (1.11 to 1.65)	
Fifth quintile	0.99 (0.67 to 1.46)	1.81*** (1.49 to 2.20)	
Illness severity (Ref–healthy)			4155
Mild symptoms	1.12 (0.72 to 1.74)	0.27*** (0.23 to 0.33)	
Moderate symptoms	1.17 (0.75 to 1.83)	0.23*** (0.19 to 0.27)	
Severe/life-threatening symptoms	2.60*** (1.50 to 4.49)	0.37*** (0.27 to 0.50)	
Reason for attendance (Ref–adult seeking care)			4115
Adult accompanying adult	0.76 (0.43 to 1.35)	1.04 (0.90 to 1.35)	
Adult accompanying child	0.71** (0.54 to 0.95)	0.70*** (0.62 to 0.73)	
Previously sought care (Ref–No)			
Yes	1.82*** (1.40 to 2.38)	0.56*** (0.49 to 0.64)	4097
Referral (Ref–No)			
Yes	2.88*** (2.10 to 3.94)	2.76*** (2.30 to 3.30)	4099
Facility type (Ref–health centre)			4115
Community hospital	0.42** (0.28 to 0.61)	1.48*** (1.25 to 1.76)	
District hospital	0.47*** (0.29 to 0.76)	0.42*** (0.31 to 0.56)	
Tertiary hospital	1.43** (1.06 to 1.93)	2.20*** (1.84 to 2.62)	
Facility ownership (Ref–governmental)			
Non-governmental	0.69** (0.51 to 0.92)	1.36*** (1.19 to 1.56)	4115
Facility location (Ref–urban)			
Rural	0.64*** (0.48 to 0.85)	0.50*** (0.43 to 0.59)	4115
Service area (Ref–other clinics/outpatients)			4115
Disease clinics	1.70** (1.19 to 2.44)	2.12*** (1.73 to 2.60)	
Emergency department	2.20 (0.83 to 5.87)	3.28*** (1.89 to 5.69)	
Maternity/gynaecology inpatient	1.32 (0.56 to 3.14)	9.30*** (6.63 to 13.02)	
Maternity/gynaecology outpatient	0.80 (0.45 to 1.40)	2.87*** (2.30 to 3.57)	
Other inpatient wards	0.74 (0.37 to 1.48)	2.04*** (1.55 to 2.70)	
Surgery	6.23*** (2.55 to 15.25)	6.00*** (3.12 to 11.53)	
Any inpatient days (Ref–No)			
Yes	0.58*** (0.39 to 0.87)	2.51*** (2.16 to 2.91)	4115
Travel time to facility (10-minute units)	1.04*** (1.01 to 1.06)	0.99 (0.98 to 1.01)	4057
Time spent with HCW (10-minute units)	0.99 (0.92 to 1.07)	1.17*** (1.14 to 1.20)	3693
Accessed required medication (Ref–Yes)			4115
No	7.58*** (5.70 to 10.08)	0.12*** (0.07 to 0.20)	
None prescribed	0.66 (0.30 to 1.44)	1.41** (1.11 to 1.79)	
Any fees paid to the facility (Ref–No)			
Yes	0.28*** (0.18 to 0.45)	0.78*** (0.66 to 0.92)	4115

* *p<0.05, ***p<0.01.

HCW, healthcare worker.

### Multivariable regression analysis

Table 5 reports the adjusted associations between covariates and the primary outcome across the three models. We excluded the variable describing reason for attendance from multivariable modelling due to collinearity with age detected via variance influence factors. We note that some estimates of effect sizes are imprecise, with wide CIs, likely reflecting sparse data in certain outcome categories despite category aggregation. These findings should therefore be interpreted cautiously, with greater emphasis placed on the direction and consistency of associations rather than the precise magnitude of effect estimates.

**Table 5:** Multivariable analysis of individual and health service factors and service user-reported quality of care

Service user-reported quality of care adjusted relative risk ratio (95% CI) (reference outcome–good)						
	Model 1		Model 2		Model 3	
	Patient and illness characteristics		Model 1+facility and visit characteristics		Model 2+additional service delivery factors	
Variables	Neutral–very bad	Very good	Neutral–very bad	Very good	Neutral–very bad	Very good
Age (10-year units)	1.092 (0.966 to 1.235)	1.083 (0.959 to 1.223)	1.051 (0.967 to 1.143)	1.060 (0.936 to 1.200)	1.100** (1.018 to 1.188)	1.053 (0.941 to 1.178)
Sex (Ref–female)						
Male	1.141 (0.876 to 1.484)	1.122 (0.932 to 1.349)	1.158 (0.891 to 1.505)	1.236** (1.048 to 1.456)	1.219 (0.954 to 1.558)	1.218** (1.029 to 1.442)
Education level (Ref–no education/some primary)						
Primary/some secondary	0.666*** (0.492 to 0.900)	1.068 (0.780 to 1.461)	0.714** (0.525 to 0.970)	1.062 (0.796 to 1.418)	0.661** (0.467 to 0.935)	1.051 (0.806 to 1.371)
Secondary/tertiary	0.718 (0.491 to 1.051)	1.669** (1.046 to 2.664)	0.780 (0.537 to 1.135)	1.372 (0.906 to 2.076)	0.772 (0.483 to 1.236)	1.504** (1.020 to 2.217)
Wealth quintile (Ref–first quintile)						
Second quintile	0.996 (0.663 to 1.497)	1.141 (0.818 to 1.591)	0.963 (0.650 to 1.425)	1.050 (0.762 to 1.445)	1.032 (0.579 to 1.841)	0.981 (0.700 to 1.376)
Third quintile	0.954 (0.583 to 1.560)	1.185 (0.807 to 1.741)	0.990 (0.586 to 1.672)	1.103 (0.793 to 1.533)	1.051 (0.539 to 2.047)	0.945 (0.692 to 1.291)
Fourth quintile	1.103 (0.703 to 1.729)	1.143 (0.702 to 1.860)	1.107 (0.707 to 1.732)	0.917 (0.584 to 1.440)	1.240 (0.730 to 2.107)	0.787 (0.525 to 1.181)
Fifth quintile	1.120 (0.609 to 2.060)	1.482 (0.841 to 2.612)	0.990 (0.559 to 1.755)	1.104 (0.677 to 1.801)	1.139 (0.686 to 1.892)	0.912 (0.562 to 1.479)
Illness severity (Ref–healthy)						
Mild symptoms	1.071 (0.625 to 1.834)	0.263*** (0.143 to 0.482)	1.299 (0.713 to 2.366)	0.279*** (0.154 to 0.505)	0.870 (0.437 to 1.731)	0.345*** (0.178 to 0.672)
Moderate symptoms	1.162 (0.689 to 1.961)	0.200*** (0.121 to 0.332)	1.572 (0.840 to 2.940)	0.237*** (0.128 to 0.441)	1.355 (0.638 to 2.880)	0.283*** (0.138 to 0.584)
Severe/life-threatening symptoms	2.773*** (1.408 to 5.462)	0.326*** (0.158 to 0.672)	3.993*** (1.868 to 8.532)	0.442** (0.203 to 0.964)	2.148 (0.936 to 4.929)	0.508 (0.205 to 1.262)
Previously sought care (Ref–No)						
Yes	1.779** (1.120 to 2.824)	0.525*** (0.359 to 0.769)	1.462 (0.963 to 2.219)	0.530*** (0.356 to 0.789)	1.535*** (1.109 to 2.125)	0.518*** (0.344 to 0.780)
Referral (Ref–No)						
Yes	2.871** (1.010 to 8.156)	2.893 (0.892 to 9.383)	3.156*** (1.589 to 6.271)	1.514 (0.988 to 2.319)	2.418*** (1.416 to 4.128)	1.444** (1.002 to 2.080)
Facility type (Ref health centre)						
Community hospital			0.403*** (0.214 to 0.761)	1.399 (0.506 to 3.863)	0.530** (0.299 to 0.939)	1.157 (0.364 to 3.682)
District hospital			0.278** (0.0861 to 0.896)	1.066 (0.152 to 7.460)	0.379 (0.123 to 1.163)	1.124 (0.190 to 6.667)
Tertiary hospital			0.596 (0.161 to 2.209)	4.271** (1.027 to 17.77)	1.258 (0.448 to 3.527)	4.060 (0.851 to 19.36)
Facility ownership (Ref–governmental)						
Non-governmental			0.757 (0.344 to 1.664)	3.677** (1.100 to 12.28)	1.570 (0.728 to 3.388)	5.819*** (1.550 to 21.84)
Facility location (Ref–urban)						
Rural			0.668 (0.330 to 1.349)	0.803 (0.343 to 1.880)	0.665 (0.322 to 1.374)	0.738 (0.281 to 1.938)
Service area (Ref–other clinics/outpatients)						
Disease clinics			1.712** (1.064 to 2.752)	1.547 (0.916 to 2.610)	1.375 (0.793 to 2.383)	1.456 (0.906 to 2.341)
Emergency department			1.402 (0.553 to 3.554)	1.191 (0.404 to 3.515)	1.039 (0.452 to 2.390)	1.065 (0.365 to 3.105)
Maternity/gynae inpatient			2.738** (1.029 to 7.284)	2.779 (0.913 to 8.465)	2.085 (0.819 to 5.310)	2.129 (0.594 to 7.622)
Maternity/gynae outpatient			1.185 (0.703 to 1.998)	1.089 (0.587 to 2.020)	1.318 (0.730 to 2.380)	1.116 (0.546 to 2.280)
Other inpatient ward			0.548 (0.275 to 1.094)	0.540 (0.164 to 1.776)	0.470 (0.173 to 1.277)	0.326 (0.0950 to 1.121)
Surgery			2.749*** (1.311 to 5.762)	2.984*** (1.511 to 5.894)	1.885** (1.059 to 3.357)	2.979*** (1.614 to 5.498)
Any inpatient days (Ref–No)						
Yes			0.502 (0.203 to 1.238)	2.721 (0.930 to 7.959)	0.974 (0.426 to 2.224)	2.721 (0.944 to 7.842)
Travel time to facility (10-minute units)					1.022 (0.998 to 1.047)	1.000 (0.973 to 1.028)
Time spent with HCW (10-minute units)					1.005 (0.908 to 1.114)	1.078 (0.977 to 1.189)
Accessed required medication (Ref–Yes)						
No					7.785*** (4.735 to 12.780)	0.176*** (0.111 to 0.279)
None prescribed					1.102 (0.448 to 2.707)	0.907 (0.528 to 1.557)
Any fees paid to the facility (Ref–No)						
Yes					0.362*** (0.177 to 0.741)	0.661 (0.341 to 1.278)
Constant	0.0485*** (0.0226 to 0.104)	1.164 (0.495 to 2.735)	0.0968*** (0.0429 to 0.218)	0.367 (0.0787 to 1.709)	0.0364*** (0.00979 to 0.135)	0.334 (0.0502 to 2.229)
Observations	4065	4065	4065	4065	3594	3594

CI in parentheses.

* *p<0.05, ***p<0.01.

HCW, healthcare worker.

In model 1, age, sex and wealth quintile were not found to be associated with perceptions of care quality. Illness severity, having previously sought care, and referral were associated with respondents having a more negative perception of quality of care. Importantly, participants who were referred from another facility were nearly three times more likely to perceive care negatively (adjusted relative risk ratio (ARRR) 2.87, 95% CI 1.01 to 8.16, p=0.048). Compared with participants who had completed none or some primary education, those who had completed primary/some secondary education were less likely to perceive care negatively. Participants with secondary/tertiary education were associated with having a more positive perception of quality of care.

Within model 2, all facility and visit covariates, except facility location and any inpatient days, were associated with the primary outcome. Compared with those interviewed at health centres, individuals interviewed at tertiary hospitals were over four times more likely to perceive care positively (ARRR 4.27 (95% CI 1.04 to 17.77), p=0.046), while those at non-governmental facilities, compared with governmental facilities, were 3.7 times more likely (ARRR 3.68 (95% CI 1.10 to 12.28), p=0.035) to do so. Male sex was now found to be significantly associated with positive perceptions of care quality. Only primary/some secondary education level was significantly associated with less negative perceptions. While those who had previously sought care remained less likely to report very positive perceptions, the association with negative perceptions was no longer statistically significant.

Model 3 represents the final adjusted model including all covariates. In this model wealth quintile, facility location, any inpatient days, travel time to the facility and time spent with HCW were not associated with the primary outcome. Sex, education level (secondary/tertiary vs no education/some primary), illness severity (mild symptoms, moderate symptoms vs healthy), having previously sought care, referral, non-governmental facility ownership and service area (surgery vs other outpatients) were associated with respondents having a more positive perception of care quality. Service users who received care in a non-governmental facility were nearly six times more likely to perceive quality of care positively (ARRR 5.82 (95% CI 1.55 to 21.84), p=0.009). Age, education level (primary/some secondary vs no education/some primary), having previously sought care, referral, facility type (community hospital vs health centre), access to required medication (no vs yes) and paying any fees to the facility were found to be associated with respondents having a negative perception of care quality. Service users who could not receive prescribed medication were nearly eight times (ARRR 7.79 (95% CI 4.74 to 12.78), p=0.000) more likely to have a negative perception of care quality compared with those who could. Similarly, service users who could not access prescribed medication were much less likely to perceived care as being very good or better (ARRR 0.18 (95% CI 0.11 to 0.28), p=0.000).

Results of sensitivity analysis conducted using multiple imputation by chained equations are available in online supplemental file 1, table S4. Across the three models, results from the sensitivity analysis were broadly consistent with the analysis reported in table 3 in terms of direction and size of effect.

Regression analyses exploring the relationships between covariates and perceptions of quality relating to HCW consultation, respect, waiting time and treatment availability are presented in online supplemental file 1, tables S5–S8. In all models, we observed that sex, illness severity, previously seeking care, referral, facility ownership, service area, access to required medication and any fees paid to the facility were significantly associated with perceptions of care quality with variation is the size of the effect or associated category. We observed frequent differences between these models and model 3 (table 5) when examining associations with patient and illness characteristics. For example, age was not significantly associated with the perceptions of care quality in any of the other models, education level was only associated perceptions of quality relating to waiting time, and wealth was found to be associated with perceptions of quality relating to HCW consultation and treatment availability.

## Discussion

Empirical evaluation of how service users, the consumers of healthcare services, perceive the quality of the care they receive is central to improving system performance.^4^ Our study explores how over 4000 service users in Malawi rate quality of care at 30 facilities across the country and the associations between key individual-level and health service determinants and reported quality. We found that quality of care was reported as being high across all evaluated dimension and identified several important determinants of perceived quality related to facility ownership and service delivery factors such as the availability of prescribed medication and fees paid to the facility.

Our findings contribute to the existing academic literature from Malawi which describes both moderate to low service user-reported quality of primary care services,^23 24^ specific facilities^29^ or interpersonal care delivered during labour,^25^ alongside evidence of high or very high perceptions of health service quality.^21 22 26–28 37^ While variation in these prior results is likely due to the breadth of methods applied, services evaluated and timing of evaluations, our study offers a contemporary, national, multiservice and multifacility evaluation in which over 90% of respondents reported that the overall quality of care they, or those they accompanied, received was ‘good’ or ‘very good’. Comparatively, nationally representative evidence from Malawi, in which providers adherence to, or knowledge of clinical guidelines, was assessed, suggests quality of care across many services is low^6 38^ despite substantial policy focus on quality improvement. While participant’s in our study were more likely to perceive quality related to waiting times and the availability of treatment negatively, likely associated with historic constraints on resources for healthcare impacting the availability of essential medical consumables^39 40^ and trained healthcare professionals,^16^ we highlight the disparity between data derived from objective measures and subjective user-reported assessment of quality presented here. This is possibly driven by ‘consumers’ expectations of healthcare.^41 42^ Service users across several LMICs were found to assess objectively poor quality care as being good or better, when accounting for low technical awareness of correct treatment.^41^ Importantly, service users with higher expectations are found to be more ‘active’ in seeking and receiving higher quality health services^43 44^ highlighting potential to improve health outcomes through increasing expectations. However, we note that in our study the expectations of service users were not measured meaning we were not able to explicitly evaluate the association between users’ expectations and reported quality.

Additionally, we identified a wide range of patient and healthcare characteristics that are associated with service user-reported quality in this population through regression analyses. At the individual level, several sociodemographic characteristics and self-perceived illness severity were found to be associated with the primary outcome, in line with the existing literature from beyond Malawi.^35^ Importantly, we found that the inability for service users to access prescribed treatment, compared with those who could, was the single largest predictor of rating care negatively. Evidence suggests the availability of key medicines across the Malawian health system varies substantially by system level, facility type and ownership^39 40^ with Mohan *et al* identifying a mean availability for a subset of key medicines of just over 50% across 940 health facilities.^39^ Given our results, and that limited availability of medicines can impact health outcomes and drive out-of-pocket costs,^45^ concerted focus on policies to improve consumable availability, such as improvements to both access and management of drugs in lower level facilities,^39^ could lead to improvements in reported care quality and health.

Beyond medication availability, we found participants were more likely to rate care as ‘very good’ compared with ‘good’ if attending a non-governmental facility for care. Higher reported quality of care for those receiving treatment at Christian faith-based providers in Africa has been well documented^46 47^ aligning with our own findings as CHAM facilities constituted most non-governmental facilities in the sample (38% of all facilities included, 85% of non-governmental facilities). This effect was even more pronounced in regression analyses in which perceived quality relating to waiting times was the primary outcome (online supplemental file 1, table S7), suggesting that reduced waiting times in non-governmental facilities may be associated with more positive self-reported quality. Additional reasons for higher perceived quality of faith-based providers are unclear but may be due to delivery of more compassionate interpersonal care and the relationship between religion and organisational culture.^46^ However, evidence from Malawi suggests that for sexual and reproductive healthcare, service users at CHAM facilities are less likely to receive services related to prevention and investigation of sexually transmitted infections or promotion of condom use^48^ suggesting despite higher perceived quality there are important shortcomings in service availability.

While our study provides important insights into service user-reported quality of care in Malawi, there are some limitations. Participants who opted to undertake the exit interview may have some important differences compared with the wider care-seeking population as over two thirds of our sample were receiving outpatient care and because data from patients who declined to participate in the study or died during care was understandably never captured. Additionally, while data collectors provided all participants with clear information about the study and independence between the research team and facility management, it is possible some participants may have overestimated quality for fear of repercussions associated with lower ratings. Another key limitation is that while we did collect data relating to why service users initially sought care this data was of very mixed quality and ranged from symptom-based complaints to confirmed diagnoses meaning we could not explore the impact of health condition on perceived quality, beyond reported severity. This also meant that while some self-reported care processes were captured in our dataset (eg, tests delivered, consultation delivered), we lacked both the clinical detail and guideline-based standards needed to assess whether care was appropriate to clinical need. We therefore could not determine whether patients received objectively high-quality care and compare that to service users’ perceptions.

In summary, our study has characterised how service users in Malawi perceive the quality of healthcare services they receive across the country. While, generally, quality was reported as being high this may be attributable to low expectations of care quality driven by the health system’s historic delivery of low-quality services, or unavailability of services, attributable to constrained resources. In addition to addressing gaps in service delivery, such as improving the availability of required medicines, empirical research exploring the drivers of improved perceptions of care quality in CHAM facilities, compared with government facilities, could be leveraged to improve service user-reported quality nationally.

## Supplementary material

10.1136/bmjopen-2025-115140 online supplemental file 1

10.1136/bmjopen-2025-115140 online supplemental file 2

10.1136/bmjopen-2025-115140 online supplemental file 3

10.1136/bmjopen-2025-115140 online supplemental file 4

## Data Availability

Data are available in a public, open access repository.
