# Supplementary material for: Healthcare service user-reported quality of care in Malawi: a national multifacility cross-sectional study
Source: BMJ Open. 2026 Jun 30;16(6):e115140. doi: 10.1136/bmjopen-2025-115140 (PMC13331198; doi:10.1136/bmjopen-2025-115140)
Supplement: Supplementary file 5 [file bmjopen-2025-115140.reviewer_comments.pdf]

PEER REVIEW HISTORY

BMJ Open publishes all reviews undertaken for accepted manuscripts. Reviewers are asked to complete a checklist review form and are provided with free text boxes to elaborate on their assessment. These free text comments are reproduced below.

ARTICLE DETAILS

Title (Provisional)

Healthcare service user-reported quality of care in Malawi: a national multi-facility cross-sectional study

Authors

Collins, Joseph H; Tafesse, Wiktoria; Chitsulo, Precious; Hallett, Timothy B; Janoušková, Eva; Mfutso-Bengo, Joseph; Mnjowe, Emmanuel; Mohan, Sakshi; Mulwafu, Watipaso; Mwapasa, Victor; Nkhoma, Dominic; She, Bingling; Suarez, Mariana; Colbourn, Timothy

---

VERSION 1 - REVIEW

**Reviewer**	**1**
**Name**	**Vanhamel, Jef**
**Affiliation**	**University of the Witwatersrand Johannesburg, School of Public Health**
**Date**	**02-Feb-2026**
**COI**	**None**

General:

Thank you very much for allowing me to review this interesting and overall well-written article. The study addresses an important policy-relevant topic and benefits from a large, national, multi facility dataset. However, I think there are some issues that warrant consideration by the authors. I have tried to point the most important ones out below, and I hope they can be useful in revising the manuscript.

Abstract:

1. If word count allows, it would be good to include some key methodological information around sampling of study sites and recruitment of participants in the abstract section.

2. Under “strengths and limitations of this study”, the second bullet point could clarify whether the mentioned association with quality of care were in the positive or negative direction. The third bullet point – talking about limitations – says the study population might

be different from those seeking care. I am not sure I understand what this means; did the interviewed participants not seek care on their own?

Introduction:

3. The paper juggles with different terms to refer to subjective measures of quality of care, including “service user satisfaction”, “patient experience of care”, and “perceived quality of care”. Although interrelated, these can all be seen as distinct constructs, yet at times they appear to be conflated or are used interchangeably. I feel that they authors could spend some more time distinguishing between “satisfaction”, “perceived quality”, and “patient experiences of care”, and justify treating these as equivalent, or else, justify and clearly explain the focus on perceived quality of care specifically. As their data are based on exit interviews, it seems to me that patients’ responses are unavoidably based on their last experience (rather than perception?).

4. The authors argue (p.3 line 27-28) that perceived quality is a valid proxy for overall quality, yet later (in the discussion) they attribute high scores to low expectations of quality of care. This creates some tension. If perceptions are expectation-driven, their validity as a quality measure is weakened. This could be acknowledged earlier or dealt with a bit more elaborately in the discussion.

Methods:

5. The measurement instrument (survey) is not described in sufficient detail. There is no information on prior validation, psychometric properties, or internal consistency. It is unclear whether items were adapted from existing tools or developed from scratch.

6. Sampling of patients: how did selecting “every Xth patient exiting the facility” work in practice; were enumerators stationed at different exits (if applicable)? Who keeps counting when surveys are ongoing? How to distinguish between patients and visitors/staff? There seems to be large room for error here... Relatedly, who would people “accompanying someone who was discharged” be? Was this largely people accompanying minors? And how were their responses treated differently in the analysis (i.e. these might be systematically different from actual patients undergoing the care)?

7. Translation seems to have been done ad hoc by enumerators during interviews. This raises concerns about standardisation and measurement error. There is no mentioning of back-translation or piloting of translated items.

8. How long – on average – did the survey take to complete, and was it exclusively enumerator-administered or was there also a self-administered option? The latter could be a measure to reduce social desirability bias further due to increased privacy and sense of anonymity.

9. The primary outcome collapses five Likert responses into three categories, merging “Neutral”, “Bad”, and “Very bad”. This combines conceptually distinct responses as a neutral response does not necessarily indicate poor quality. Although I understand the small

numbers in “Bad/Very Bad” categories, I was wondering if some sensitivity analyses were done with alternative ways of merging the outcome categories.

10. Given the ordered nature of the response categories of the outcome variable, would multinomial regression (assuming unordered categories) be the best approach for associative analysis? Did the authors consider a multilevel ordered logistic regression model, or was this deemed inappropriate for some reason?

11. The incremental modelling strategy is presented as addressing confounding, but some variables added in later models may be mediators. For example, medication availability and fees paid may lie on the causal pathway between facility type and perceived quality. This complicates interpretation of adjusted associations. In addition, were all variables included in the different models, or only those found to be statistically significant in bivariable analysis (and if so, at which level of significance)?

12. Excluding cases with missing covariates assumes data are missing completely at random. The authors test clustering of missingness by facility only. Was assessment of missingness by other key individual characteristics not deemed necessary?

Results:

13. The mean age of 25.7 years with a very large standard deviation suggests a sample consisting mainly of young adults, yet perceptions are often reported as being more homogeneous. Maybe this could receive a bit more emphasis in the discussion?

14. Is there any information about refusals to being interviewed (e.g. among those patients that were approached)? Or did all patients that were approached agree to be interviewed? This would provide further insights into the potential for sampling/selection bias (e.g. maybe those who refused to participate systematically had other perceptions of care)? For instance, fewer people were interviewed from district hospitals, but with the described sampling approach this seems strange, suggesting maybe more people refused to participate here?

15. It says 6.2% of the sample rated the quality as “neither good nor bad, bad or very bad”; however, one would expect this to be 7.7% (as 92.3% was at the counterpart level of “very good or good”).

16. Throughout the article, I would suggest to consistently include p-values in the tables and also include numbers consistently in the main manuscript for every statistically significant association mentioned (e.g. aRRR=XX; 95%CI=XX-XX; p=<XX). This makes it easier for the reader, not having to go look-up every element in the different tables. Also, usually odds ratios are reported for logistic regression, is there any reason the authors reported relative risk ratios?

17. As mentioned before, in Model 2, was everything adjusted for everything, or what approach was applied to adjust for confounders? In a way it is odd that the association with sex now changes, as the variables added to this model cannot influence sex (which is a rather fixed individual characteristic).

18. In Model 3, sex is mentioned as a positive association with quality of care, but was it being male or female? Similarly, age was mentioned as associated with negative perception of quality of care; was this increasing age?

19. There are some very large effect sizes with wide confidence intervals, especially for facility ownership and tertiary facilities, suggesting sparse data in some categories? This should prompt caution in interpretation and possibly category aggregation. Maybe the authors want to respond to this.

Discussion:

20. The discussion occasionally implies causal relationships despite the cross-sectional design. Statements suggesting that improving medicine availability “would likely drive improvements” in perceived quality go beyond the evidence.

21. The explanation that high reported quality reflects low expectations is plausible, but largely speculative. The paper does not measure expectations directly so this argument relies heavily on external literature and should maybe be framed more cautiously.

22. The contrast between subjective quality and objective quality measures is mentioned a few times in the paper. However, no objective quality indicators from the same dataset are analysed alongside perceptions. This limits the strength of the comparison here and could maybe be mentioned as limitation or area for further research.

23. The limitations section lacks some reflection on possible measurement limitations (e.g. lack of standardised tool [or if it was standardised, need to mention it in text], possible translation issues) and could contain some more information on possible systematic errors caused by refusal to participate among patients (see earlier).

**Reviewer**	**2**
**Name**	**Grinberg, Keren**
**Affiliation**	**Ruppin Academic Center, Nursing Sciences Department**
**Date**	**05-Feb-2026**
**COI**	**None**

Thank you very much for the opportunity to review an interesting and important article on the subject of " Healthcare service user-reported quality of care in Malawi: a national multi-facility cross-sectional study ". This manuscript reports a national multi-facility cross-sectional study using service-user exit interviews (n=4,181) collected between January- May 2024 across 30 facilities in 15 districts in Malawi. The authors describe overall perceived quality of care and assess associations between patient/service characteristics and perceived quality using multinomial logistic regression.

Overall ratings were high (about 57.6% “Good” and 34.7% “Very good”), with more negative ratings concentrated in specific dimensions such as waiting times and treatment availability.

Several suggestions for corrections / additions to be made:

• The primary outcome collapses responses into three categories (“Very good”, “Good”, and a combined “Neutral -Very bad”). Please justify combining neutral with negative responses, as this may mask meaningful differences and potentially distort associations (especially if “neutral” is common and conceptually distinct). Consider a sensitivity analysis using an ordinal model (if the original response scale is ordered) or alternative categorisations.

• The survey was coded in English and translated “as required during data collection” by enumerators into Chichewa/Tumbuka. This approach can introduce interviewer-level variability. Please clarify whether standard translated scripts were used, whether any back-translation/verification occurred, and what QA measures ensured consistent phrasing across teams.

• Participant selection was based on daily patient load with “every Xth person” interviewed to reach target numbers, and there were no eligibility restrictions. However, the manuscript should report numbers approached/refused (or a response rate) to help assess participation bias. The discussion notes that those opting in may differ and that patients who died during care were not captured.

• Missingness is reported in several variables (including the outcome), and complete-case exclusion was used for regression. Please provide a clear table of missingness by variable and consider multiple imputation (or at minimum, sensitivity analyses comparing complete-case vs. alternative strategies), particularly if missingness is non-trivial for key covariates.

• It is unclear whether analyses aim for nationally representative estimates and, if so, whether weights/design effects were applied. Please clarify the intended estimand and how the sampling strategy is accounted for in descriptive estimates and regression.

**Reviewer**	**3**
**Name**	**Kelson, Zoe**
**Affiliation**	**University of Exeter, Mathematics**
**Date**	**19-Mar-2026**
**COI**	**None**

This study aimed to explore how service users in Malawi perceive the quality of the healthcare they receive across services and facilities and to investigate the association between individual and health service characteristics and perceived quality of care.

Reviewer comments:

"Descriptive statistics of service user-reported quality of care were estimated in addition to multinomial logistic regression analyses used to determine the association between patient and healthcare characteristics and perceived care quality." [Abstract]

The analytical approach is concisely described by the authors in the Abstract.

"Positive or negative perceptions of care quality were associated with age, sex, education level, illness severity, previously seeking care, referral, facility type, non-governmental facility ownership, service area, access to medication and payment of fees." [Abstract]

Can the authors please clarify which factors are associated with positive or negative associations in the Abstract?

Can statistical outcomes please be reported here too?

"national multi-facility cross-sectional study using service user exit interview data... 30 health facilities across 15 districts in Malawi... 4,181 respondents surveyed after completing their visit and exiting healthcare facilities between January and May 2024" [Abstract]

and

"part of the Thanzi La Mawa (TLM) study - a cross-sectional and mixed methods study intended to improve understanding of healthcare delivery in Malawi and to inform Malawi's first wholehealth system mathematical model (the Thanzi La Onse model (TLO))"

and

"The service user exit-survey component of the TLM study was conducted at 30 health facilities across 15 districts in Malawi namely Blantyre, Chiradzulu, Dedza, Karonga, Likoma, Lilongwe, Mzimba, Mzuzu, Neno, Nkhata Bay, Nsanje, Ntcheu, Rumphi, Thyolo and Zomba."

Can the authors please further comment on the completeness and coverage of this data?

"The study design and data-collection processes have been previously described (30)."

Can the survey design please be described here? Were sample weights applied to adjust for the design?

"Data analysed for this study was collected primarily through service user exit-surveys and we have used the STROBE guidelines on cross-sectional studies (31) to report these results (Supplementary File 1 – Table S1)"

Thanks for providing a copy of the STROBE checklist.

"Data was collected between January and May 2024 at 30 health facilities including all four central (tertiary) hospitals (Queen Elizabeth, Zomba, Kamuzu, Mzuzu) and Zomba mental hospital. To ensure a representative sample 25 facilities were selected visa stratified random sampling. Facilities were selected using a random number generator in Microsoft Excel from the National Master Health Facility Register following stratification by facility type (health centre, community hospital, secondary hospital), patient volume (relatively lower or higher),

ownership (MOH or CHAM), and location (urban or rural). A full list of included facilities is available in Supplementary File 1 – Table S2"

and

"Enumerators randomly selected potential participants from adults exiting the facility after being discharged or accompanying someone who was discharged. To survey a representative random sample of patients, participant selection was based on daily patient load (estimated via facility registers or waiting room attendance). Given enumerators aimed to survey 15 patients per day, every Xth patient was interviewed to reach this number of surveys (e.g. if 60 people were waiting for treatment, every 4th person was interviewed)."

and

"In total, data was collected from 4,181 respondents across all included facilities"

Can the authors please assess whether representativeness has been achieved?

"The service user exit-survey, available in Supplementary File 2, consisted of possible 68 questions across seven areas; patient details (11 questions), facility visit (14 questions), patients' access to medicines (2 questions), patients' persistence in care-seeking (3 questions), costs and opportunity costs of attending facility (11 questions), household assets (17 questions) and perceptions of quality of care (10 questions)."

Thanks for providing a copy of the survey questions.

"Characteristics of the sample, the included facilities, and the service user-reported quality of care have been described first with descriptive statistics. "

Can the authors please specify the type of statistical descriptor(s) used here, and assess the distributional assumptions of the data (e.g. normality)?

"The association between variables and the primary outcome, overall service user-reported quality of care for the facility visit, was explored via multinomial logistic regression."

and

"We developed three multivariable models, each incrementally incorporating additional variables to systematically address confounding"

An appropriate modelling methodology has been applied by the authors.

"The primary outcome of this analysis was a categorical variable derived from the Likert scale survey statement assessing 'overall' quality of the participants visit, with three responses: 'Very good', 'Good', 'Neutral - Very bad'. Using those who reported that care was 'Good' as the reference category allowed for focus of analytical power on the extremes of responses and to account for the small number of respondents who rated overall quality as 'Bad' or 'Very bad'."

Can the authors please consider exploring an ordinal regression analysis, keeping the outcome variable as multiple distinct categories?

"Where covariates are categorical or binary variables derived from survey questions, Table S3 - Supplementary File 1 describes the relevant derivation."

To minimise loss of information, can the authors please attempt to keep variables in continuous or granular form as much as feasible in the analysis?

"Missing data was observed in six variables, including service user-reported quality of care (1.6%), and ranged from 0.4% - 10.2% of total entries. To assess if missing data in the primary outcome was clustered by facility, we conducted a Chi-square test of independence between the missingness indicator and facility."

and

"Individuals with missing outcome or covariate data were excluded from regression models"

Can the authors please comment on whether missing data can be considered to be missing at random?

Can the authors please consider a sensitivity analysis on their approach to missing data (applying multiple imputation, for instance)?

"The Stata .do analysis file is available as Supplementary File 4."

The authors transparently provide the analysis code, supporting reproducibility and further research.

"Table 2 – Sample and facility characteristics"

Can race or ethnicity please be reported in the study?

"As this study was cross-sectional, causal relationships cannot be confirmed. Additionally, service users who opted to participate may have some important differences compared to the wider care-seeking population (e.g. those who sought care, those who died during treatment)" [Strengths and Limitations of this study]

and

"Whilst our study provides insights into service user-reported quality of care in Malawi, there are some limitations. Participants who opted to undertake the exit interview may have some important differences compared to the wider care-seeking population as over two thirds of our sample were receiving outpatient care and because data from patients who died during care was understandably never captured. Additionally, while data collectors provided all participants with clear information about the study and independence between the research team and facility management, it is possible some participants may have overestimated quality for fear of repercussions associated with lower ratings. Finally, due to the wide range of reasons for attending the facility in our sample it was not possible to explore the effect of objective indicators such as adherence to clinical guidelines on

perceptions of care despite collecting data on treatment delivery (e.g. tests delivered, observations delivered, consultation etc. – see Supplementary File 2). "

A discussion on the study limitations is provided.

VERSION 1 - AUTHOR RESPONSE

Reviewer: 1
Thank you very much for allowing me to review this interesting and overall well-written article. The study addresses an important policy-relevant topic and benefits from a large, national, multi facility dataset. However, I think there are some issues that warrant consideration by the authors. I have tried to point the most important ones out below, and I hope they can be useful in revising the manuscript.

We thank the reviewer for their time and comments.

Abstract:
1. If word count allows, it would be good to include some key methodological information around sampling of study sites and recruitment of participants in the abstract section.

We have now added the following sentence to the abstract: *"A national multi-facility cross-sectional study using service user exit interview data in which all central hospitals were sampled, and remaining facilities were selected via random stratified sampling".*

2. Under "strengths and limitations of this study", the second bullet point could clarify whether the mentioned association with quality of care were in the positive or negative direction. The third bullet point – talking about limitations – says the study population might be different from those seeking care. I am not sure I understand what this means; did the interviewed participants not seek care on their own?

In response to editorial comments, bullet points 1 and 2 have now been updated. We have now amended the final bullet point to say *"Service users who opted to participate may have some important differences compared to the wider population (e.g. those who did not seek care, those who died during treatment)"*

Introduction:
3. The paper juggles with different terms to refer to subjective measures of quality of care, including "service user satisfaction", "patient experience of care", and "perceived quality of care". Although interrelated, these can all be seen as distinct

constructs, yet at times they appear to be conflated or are used interchangeably. I feel that they authors could spend some more time distinguishing between "satisfaction", "perceived quality", and "patient experiences of care", and justify treating these as equivalent, or else, justify and clearly explain the focus on perceived quality of care specifically. As their data are based on exit interviews, it seems to me that patients' responses are unavoidably based on their last experience (rather than perception?).

We agree with the reviewer that "patient satisfaction" and "patient experience of care" are distinct constructs. We however propose that there is less clear distinction between "patient user satisfaction" and "patient perceptions of quality" given that, as the reviewer proposes, expectations of care quality likely impact both measures. Our survey measures perceived quality of care across several dimensions with respondents asked explicitly about the quality of their recent interaction with the health system at facility exit (Supplementary File 2). We have opted to continue to refer to the study's outcome as "service user-reported quality of care" as we believe this sufficiently highlights that the measure is service user perceptions. This is a similar approach taken by colleagues at the QUEST network who applied the people's voice survey to assess patient-reported quality across 14 countries including several LMICs (Lewis et al., 2023).

We do note that the evidence evaluating the link between patients' perceptions of quality (excluding patient experience) and objective measures of clinical quality is more mixed than we had previously indicated. However, we believe that understanding how patients perceive the quality of care they receive is important for several reasons; (i) high-quality care is responsive to the preferences, needs and values of service users, (ii) as consumers of healthcare services users are well placed to assess the quality of a product and (iii) perceptions of care quality are linked to important health behaviours such as care seeking.

To address this comment we have rewritten the second paragraph of the introduction to draw the distinction between patient experience and other subjective quality measures, explore the overlap between satisfaction and perceived quality, and better motivate the need to explore perceptions of quality as an outcome in its own right (not as a proxy for objective clinical quality). In addition, we have reviewed the entire manuscript to ensure consistency with our initial position highlighted in the introduction.

<u>References</u>:

Lewis T, Kassa M, Kapoor N et al. User-reported quality of care: findings from the first round of the People's Voice Survey in 14 countries. The Lancet Global Health, 2023; 12, e112-e122

4. **The authors argue (p.3 line 27-28) that perceived quality is a valid proxy for overall quality, yet later (in the discussion) they attribute high scores to low expectations of quality of care. This creates some tension. If perceptions are expectation-driven, their validity as a quality measure is weakened. This could be acknowledged earlier or dealt with a bit more elaborately in the discussion.**

Please see our response to the above comment. Given that we no longer position perceived quality as a proxy for clinical quality we believe this tension has been addressed.

Methods:

5. The measurement instrument (survey) is not described in sufficient detail. There is no information on prior validation, psychometric properties, or internal consistency. It is unclear whether items were adapted from existing tools or developed from scratch.

Our survey was developed for the conduct of this study. Content and face validation of the initial survey design was conducted by the research team. Prior to final data collection, during January 2024, all survey tools used within the TLM study were piloted following initial review of the instruments with enumerators and the research team. Survey tools were initially piloted at Kabudula Community Hospital in Lilongwe, after which they were reviewed and refined, followed by a second pilot at Area 25 Health Centre in Lilongwe. Further detail about the survey, piloting and validation process have been added to the method section.

The primary outcome of this analysis is a single global item assessing reported quality of care, which is an established approach utilised across contextually similar settings (see above - Lewis et al). Because our outcome is a direct report of quality (not a latent variable derived from measures of an underlying construct) measures of internal consistency such as Cronbach's alpha would not be applicable here. We would note that domain specific quality ratings (healthcare worker consultation, respect, treatment availability, waiting time) were positively correlated with overall quality ($p=0.000$), providing evidence of criterion validity.

6. Sampling of patients: how did selecting "every Xth patient exiting the facility" work in practice; were enumerators stationed at different exits (if applicable)? Who

keeps counting when surveys are ongoing? How to distinguish between patients and visitors/staff? There seems to be large room for error here…

Enumerators were positioned at either the gate which marked the exit/entrance of a given facility (of which there is only one per facility) or at the pharmacy where discharged patients would be collecting their prescriptions. Our survey protocol was to survey the Xth patient based on load, in practice, because there was only one enumerator collecting data at a given facility, identification of the Xth patient was challenging and instead random selection of patients based on the recruitment target for a given facility was relied on. This meant that roughly every 15-20 minutes (the length of time taken to conduct a survey) a new participant was approached for data collection which is equivalent to every Xth patient leaving the facility assuming a constant flow of patients.

We have now updated the methods section to reflect this.

Relatedly, who would people "accompanying someone who was discharged" be? Was this largely people accompanying minors? And how were their responses treated differently in the analysis (i.e. these might be systematically different from actual patients undergoing the care)?

As mentioned in the methods, survey respondents were either the service users themselves or people accompanying service users to receive care/at discharge. This is captured in the variable 'reason for attendance', which categorises respondent's reason for attending facility (adult seeking care, adult accompanying a child, adult accompanying an adult) (Tables 1 and 2). The reviewer will see from Table 2 that nearly two thirds of the respondents were adults who had sought care for themselves. As stated in the results section, in bivariate analysis reason for attendance was associated with the primary outcome (Table 3) however was not included in multivariable models due to collinearity with age.

7. Translation seems to have been done ad hoc by enumerators during interviews. This raises concerns about standardisation and measurement error. There is no mentioning of back-translation or piloting of translated items.

We apologies for any confusion relating to translation of the service. Firstly, we note that enumerators report that most surveys were delivered in English. Additionally, prior to the data collection the survey was translated into Chichewa and submitted for review as part of the studies ethics application. During initial training and piloting phase enumerators were further guided on translation. To reduce the risk of measurement

error, several key terms were pre-translated into Tumbuka prior to data collection within Kobo Toolbox.

This information has been added to the methods section.

8. How long – on average – did the survey take to complete, and was it exclusively enumerator-administered or was there also a self-administered option? The latter could be a measure to reduce social desirability bias further due to increased privacy and sense of anonymity.

On average the survey took 15-minutes to complete and was exclusively enumerator-administered. This information has been added to the methods section.

We appreciate self-administration may reduce bias, we had previously noted that social desirability may impact results in our limitation section - *"Additionally, while data collectors provided all participants with clear information about the study and independence between the research team and facility management, it is possible some participants may have overestimated quality for fear of repercussions associated with lower ratings."*

9. The primary outcome collapses five Likert responses into three categories, merging "Neutral", "Bad", and "Very bad". This combines conceptually distinct responses as a neutral response does not necessarily indicate poor quality. Although I understand the small numbers in "Bad/Very Bad" categories, I was wondering if some sensitivity analyses were done with alternative ways of merging the outcome categories.

Given the small number of respondents in the "Neutral", "Bad" and "Very Bad" categories we are limited in how we can configure our primary outcome categories. We believe that the approach we have taken, given the structure of the data, affords a more detailed analysis than the only other alternative configuration we could use (binary outcome comparing "Very Bad/Bad/Neutral" with "Good/Very Good"). Our primary analysis has allowed us to present which factors are associated both with very high perceptions of quality and lower perceptions of quality without any assumptions that the associations would be the same in direction/size.

10. Given the ordered nature of the response categories of the outcome variable, would multinomial regression (assuming unordered categories) be the best approach for associative analysis? Did the authors consider a multilevel ordered logistic regression model, or was this deemed inappropriate for some reason?

Please see our above response. Given the small numbers in "Neutral", “Bad” and “Very Bad” categories an ordinal logistic regression would not be appropriate as estimates for covariates would be unstable. Additionally, we did not believe that the core assumption of ordinal regression models (proportional odds) would hold in this case. The assumption implies that covariates have a constant effect across all ‘thresholds’ for the ordinal outcome which we believe is highly unlikely in this context given the different mechanisms that likely drive perceptions of poor care vs perceptions of high-quality care. Indeed this is also borne out in our multinomial logistic regression results (as now formally tested, as explained below).

In response to your comment we have now attempted to formally test the proportional odds assumption. However, when using the initial Likert response outcome, the ordered logit models used for assumption tests failed to converge, likely due to the sparse responses in key outcome categories. We therefore did not interpret these tests for this outcome. This does however support our decision to collapse the primary outcome before modelling. For completeness, we evaluated whether using an ordered logit model would be appropriate with the collapsed primary outcome used within our primary analysis, but formal tests (e.g. Brant) indicated that the proportional odds assumption was violated.

We have updated the methodology section to document this testing and have updated the stata code in Supplementary File 4.

11. The incremental modelling strategy is presented as addressing confounding, but some variables added in later models may be mediators. For example, medication availability and fees paid may lie on the causal pathway between facility type and perceived quality. This complicates interpretation of adjusted associations. In addition, were all variables included in the different models, or only those found to be statistically significant in bivariable analysis (and if so, at which level of significance)?

Our analysis was not designed to estimate the independent causal effect of any single exposure on the primary outcome. Instead, we used incremental covariate adjustment to examine how associations changed as theoretically grouped variables were introduced. Covariates were therefore included as part of a structured explanatory model, rather than solely as confounders requiring statistical control. We acknowledge that some variables may lie on causal pathways between upstream characteristics and the outcome and therefore do not interpret coefficients from fully adjusted models as estimates of total causal effects. Rather, the models are interpreted descriptively. Variable selection was guided by theoretical relevance and previous research rather than statistical significance in bivariate analyses.

In response to reviewer feedback, we have revised the manuscript to clarify this modelling approach, including removing specific references to confounding and updating the methods section accordingly.

12. Excluding cases with missing covariates assumes data are missing completely at random. The authors test clustering of missingness by facility only. Was assessment of missingness by other key individual characteristics not deemed necessary?

We agree with the review that our evaluation of missingness within our study could be more comprehensive, beyond the reporting of missingness in Table 2 and 3.

We have now evaluated the predictors of complete case inclusion (e.g. participants with complete data) using logistic regression (please see updated stata code in Supplementary File 4). Through this process we identified that facility location is the only variable associated with complete case status (AOR 1.85, p=0.026). This effect was only significant when accounting for clustering by facility suggesting structural variation in completeness. We therefore decided to supplement our primary analysis by conducting a sensitivity analysis using multiple imputation by chained equations, the results of which are now available within the supplementary material (Supplementary Table 5). We opted not to present results from multiple imputation as our primary analysis given that the proportion of participants with complete data was high (86%) and that primary outcome data was imputed for some participants through imputation. Results from this sensitivity analysis were broadly consistent with the complete case analysis reported in Table 3 in terms of direction and size of effects.

In addition to the results of the sensitivity analysis, we have added a description of our methodological approach to addressing missingness within the methods section and a qualitative overview of this sensitivity analysis in the results section.

Results:

13. The mean age of 25.7 years with a very large standard deviation suggests a sample consisting mainly of young adults, yet perceptions are often reported as being more homogeneous. Maybe this could receive a bit more emphasis in the discussion?

The low mean age and large standard deviation in our sample is a product of the fact that we include children within our sample, that Malawi has a relatively young population and the range of ages of respondents is large. Although the survey will have been completed by those accompanying children to care, the age recorded is that of

the patient. We have now included the median age (24) and the range (0-98) within Table 2 where descriptive statistics are presented.

14. Is there any information about refusals to being interviewed (e.g. among those patients that were approached)? Or did all patients that were approached agree to be interviewed? This would provide further insights into the potential for sampling/selection bias (e.g. maybe those who refused to participate systematically had other perceptions of care)? For instance, fewer people were interviewed from district hospitals, but with the described sampling approach this seems strange, suggesting maybe more people refused to participate here?

Unfortunately we do not have information on the number of refusals although enumerators did report this was very rare. We would be limited in what inferences we could make about refusals given that information we would have about refusals would be limited to facility characteristics. We have previously highlighted that a key limitation of our study is that our sample may differ from the wider population in keyways.

Additionally, we would note that fewer interviews conducted at district hospitals is likely driven by the small number of district hospitals in the facility sample (Supplementary File 1, Table S2)

15. It says 6.2% of the sample rated the quality as "neither good nor bad, bad or very bad"; however, one would expect this to be 7.7% (as 92.3% was at the counterpart level of "very good or good").

These results refer to the total surveyed population, of which 1.5% did not answer the survey question from which the primary outcome was derived.

16. Throughout the article, I would suggest to consistently include p-values in the tables and also include numbers consistently in the main manuscript for every statistically significant association mentioned (e.g. aRRR=XX; 95%CI=XX-XX; p=)

Where results are described within the manuscript text, we have now added p values to improve clarity. We have opted to retain how p-values are indicated within the tables (***p<0.01 denoting and **p<0.05 denoting) to preserve readability given the number of results presented in each table.

17. As mentioned before, in Model 2, was everything adjusted for everything, or what approach was applied to adjust for confounders? In a way it is odd that the association with sex now changes, as the variables added to this model cannot

influence sex (which is a rather fixed individual characteristic).

Please see our response to comment 11 above. We disagree that adding an additional group of variables into the model related to facility characteristics could not impact either the size or direction of effect for the sex covariate. The emergence of an association between sex and quality after inclusion of facility-level covariates suggests that facility context may explain differences in the relationship between sex and perceived quality.

18. In Model 3, sex is mentioned as a positive association with quality of care, but was it being male or female? Similarly, age was mentioned as associated with negative perception of quality of care; was this increasing age?

We agree that there could be greater consistency with how reference categories are handled in the results tables which we have now updated in the manuscript and supplementary material.

Regarding age, this was modelled as a continuous variable scaled in 10-year increments; therefore, the reported effect size represents the relative risk ratio (RRR) associated with each additional 10-year increase in age for being in each outcome category relative to the reference outcome category.

19. There are some very large effect sizes with wide confidence intervals, especially for facility ownership and tertiary facilities, suggesting sparse data in some categories? This should prompt caution in interpretation and possibly category aggregation. Maybe the authors want to respond to this.

We believe that further aggregation of variables within the model risks combining responses which are substantially different (e.g. type of facility). Additionally, the facility ownership variable is already a product of aggregation (see Table S3).

We have now added a passage to the results section to aid interpretation of coefficients with larger effect sizes/confidence intervals.

Discussion:
20. The discussion occasionally implies causal relationships despite the cross-sectional design. Statements suggesting that improving medicine availability "would likely drive improvements" in perceived quality go beyond the evidence.

We have now amended our language to reduce implications of causality in the discussion.

21. The explanation that high reported quality reflects low expectations is plausible, but largely speculative. The paper does not measure expectations directly so this argument relies heavily on external literature and should maybe be framed more cautiously.

We have now added a sentence to that paragraph to ensure this explanation is framed more cautiously.

22. **The contrast between subjective quality and objective quality measures is mentioned a few times in the paper. However, no objective quality indicators from the same dataset are analysed alongside perceptions. This limits the strength of the comparison here and could maybe be mentioned as limitation or area for further research.**

After reviewing our discussion section we apologise for confusion around supposed 'objective' indicators captured within our data set. We understand objective measures of healthcare quality to be those in which care delivered is compared to care required based on evidence-based clinical guidelines. Within this study we did not assess objective quality of care therefore we would not be able to compare subjective and objective measures as we do not have the data to do so.

Whilst we did capture self-reported experience measures (e.g. tests delivered, consultation delivered etc) and self-reported presenting complaint (often described as symptoms) we are unable to make any inference about whether the appropriate treatment for an individual was delivered without significant assumption – something we were not willing to do. We have now clarified the point made in our limitation section to that effect.

23. The limitations section lacks some reflection on possible measurement limitations (e.g. lack of standardised tool [or if it was standardised, need to mention it in text], possible translation issues) and could contain some more information on possible systematic errors caused by refusal to participate among patients (see earlier).

Please see our response to comment 5 where we believe we have demonstrated that the tool was standardised across possible languages during the piloting phase. We don't believe this is a limitation of our study. In our limitation section we have updated the following section: *"Participants who opted to undertake the exit interview may have some important differences compared to the wider care-seeking population as over two thirds of our sample were receiving outpatient care and because data from patients*

who declined to participate in the study or died during care was understandably never captured."

Reviewer: 2
Thank you very much for the opportunity to review an interesting and important article on the subject of " Healthcare service user-reported quality of care in Malawi: a national multi-facility cross-sectional study ". This manuscript reports a national multi-facility cross-sectional study using service-user exit interviews (n=4,181) collected between January- May 2024 across 30 facilities in 15 districts in Malawi. The authors describe overall perceived quality of care and assess associations between patient/service characteristics and perceived quality using multinomial logistic regression.
Overall ratings were high (about 57.6% "Good" and 34.7% "Very good"), with more negative ratings concentrated in specific dimensions such as waiting times and treatment availability.

We thank the reviewer for their time and comments. We note that many of the reviewer's comments are similar to Reviewer 1 and we have therefore referenced our previous responses where appropriate.

Several suggestions for corrections / additions to be made:
1. The primary outcome collapses responses into three categories ("Very good", "Good", and a combined "Neutral -Very bad"). Please justify combining neutral with negative responses, as this may mask meaningful differences and potentially distort associations (especially if "neutral" is common and conceptually distinct). Consider a sensitivity analysis using an ordinal model (if the original response scale is ordered) or alternative categorisations.

Please see our response to comments 9 and 10 from Reviewer 1 where we believe we have addressed this issue.

2. The survey was coded in English and translated "as required during data collection" by enumerators into Chichewa/Tumbuka. This approach can introduce interviewer-level variability. Please clarify whether standard translated scripts were used, whether any back-translation/verification occurred, and what QA measures ensured consistent phrasing across teams.

Please see our response to comment 7 from Reviewer 1 where we believe we have addressed this issue.

3.Participant selection was based on daily patient load with "every Xth person" interviewed to reach target numbers, and there were no eligibility restrictions. However, the manuscript should report numbers approached/refused (or a response rate) to help assess participation bias. The discussion notes that those opting in may differ and that patients who died during care were not captured.

Please see our response to comments 6 and 14 from Reviewer 1 where we believe we have addressed this issue.

4. Missingness is reported in several variables (including the outcome), and complete-case exclusion was used for regression. Please provide a clear table of missingness by variable and consider multiple imputation (or at minimum, sensitivity analyses comparing complete-case vs. alternative strategies), particularly if missingness is non-trivial for key covariates.

Tables 2 and 3, in which all variables are described, include a clear indication of which variables have missing data and the proportion of missingness. Please see our response to comment 12 from Review 1 where we describe our updated approach to missingness within this study and sensitivity analysis using multiple imputation by chained equations.

5. It is unclear whether analyses aim for nationally representative estimates and, if so, whether weights/design effects were applied. Please clarify the intended estimand and how the sampling strategy is accounted for in descriptive estimates and regression.

As described in our methodology section, in addition to all data all four central hospitals and Malawi's only mental health hospital, data was collected from a sample of 25 facilities which were selected via stratified random sampling. Facilities were stratified by facility type (health centre, community hospital, secondary hospital), patient volume (relatively lower or higher), ownership (MOH or CHAM), and location (urban or rural). Our sampling frame was not powered for national representativeness. As such we have not used weights in our analysis. Importantly, the study aimed to capture variation across relevant facility contexts in Malawi. Stratified facility selection improved coverage of key service settings while random selection within strata reduced selection bias.

We have now clarified this in our methods section.

Reviewer: 3
This study aimed to explore how service users in Malawi perceive the quality of the healthcare they receive across services and facilities and to investigate the association between individual and health service characteristics and perceived quality of care.

We thank the reviewer for their time and comments. We note that many of the reviewer's comments are similar to Reviewer 1 and 2 and we have therefore referenced our previous responses where appropriate.

Reviewer comments:
"Positive or negative perceptions of care quality were associated with age, sex, education level, illness severity, previously seeking care, referral, facility type, non-governmental facility ownership, service area, access to medication and payment of fees." [Abstract]
Can the authors please clarify which factors are associated with positive or negative associations in the Abstract?
Can statistical outcomes please be reported here too?

Due to the word limit for the abstract, the number of variables associated with the primary outcome, the multinomial structure of the analysis and the varied direction of effect for each association we believe, to preserve readability, indicating which variables have an association with either better or worse perceptions of care quality is sufficient.

"national multi-facility cross-sectional study using service user exit interview data... 30 health facilities across 15 districts in Malawi... 4,181 respondents surveyed after completing their visit and exiting healthcare facilities between January and May 2024" [Abstract]
and
"part of the Thanzi La Mawa (TLM) study - a cross-sectional and mixed methods study intended to improve understanding of healthcare delivery in Malawi and to inform Malawi's first wholehealth system mathematical model (the Thanzi La Onse model (TLO))"
and
"The service user exit-survey component of the TLM study was conducted at 30 health facilities across 15 districts in Malawi namely Blantyre, Chiradzulu, Dedza, Karonga, Likoma, Lilongwe, Mzimba, Mzuzu, Neno, Nkhata Bay, Nsanje, Ntcheu, Rumphi, Thyolo and Zomba."
Can the authors please further comment on the completeness and coverage of this data?

Please see our responses to comments 9 and 10 from Reviewer 1 where we believe we address your comment about completeness. In addition, please see our response to comment 5 from Reviewer 2 where we believe we address your comment about coverage.

"The study design and data-collection processes have been previously described (30)."
Can the survey design please be described here? Were sample weights applied to adjust for the design?

Please see our response to comment 5 from Reviewer 2 where we believe we address your comment.

"Data was collected between January and May 2024 at 30 health facilities including all four central (tertiary) hospitals (Queen Elizabeth, Zomba, Kamuzu, Mzuzu) and Zomba mental hospital. To ensure a representative sample 25 facilities were selected via stratified random sampling. Facilities were selected using a random number generator in Microsoft Excel from the National Master Health Facility Register following stratification by facility type (health centre, community hospital, secondary hospital), patient volume (relatively lower or higher), ownership (MOH or CHAM), and location (urban or rural). A full list of included facilities is available in Supplementary File 1 – Table S2"
and
"Enumerators randomly selected potential participants from adults exiting the facility after being discharged or accompanying someone who was discharged. To survey a representative random sample of patients, participant selection was based on daily patient load (estimated via facility registers or waiting room attendance). Given enumerators aimed to survey 15 patients per day, every Xth patient was interviewed to reach this number of surveys (e.g. if 60 people were waiting for treatment, every 4th person was interviewed)."
and
"In total, data was collected from 4,181 respondents across all included facilities"
Can the authors please assess whether representativeness has been achieved?

Please see our response to comment 5 from Reviewer 2 in addition to our responses to your comments above.

"Characteristics of the sample, the included facilities, and the service user-reported quality of care have been described first with descriptive statistics. "
Can the authors please specify the type of statistical descriptor(s) used here, and

assess the distributional assumptions of the data (e.g. normality)?

Table 2 describes the characteristics of the sample and facilities. We include the distribution of responses for categorical variables and for continuous variables we had presented the mean and standard deviation. We have now added the median and range for these variables also. We do not believe further evaluation of the distribution of variables is required for this analysis.

"The primary outcome of this analysis was a categorical variable derived from the Likert scale survey statement assessing 'overall' quality of the participants visit, with three responses: 'Very good', 'Good', 'Neutral - Very bad'. Using those who reported that care was 'Good' as the reference category allowed for focus of analytical power on the extremes of responses and to account for the small number of respondents who rated overall quality as 'Bad' or 'Very bad'."
Can the authors please consider exploring an ordinal regression analysis, keeping the outcome variable as multiple distinct categories?

Please see our responses to comments 9 and 10 from Reviewer 1 where we believe we have addressed this issue.

"Where covariates are categorical or binary variables derived from survey questions, Table S3 - Supplementary File 1 describes the relevant derivation."
To minimise loss of information, can the authors please attempt to keep variables in continuous or granular form as much as feasible in the analysis?

We have endeavoured to keep variables in a continuous or granular form as much as is feasible for our analyses. Derivation of variables used within analyses (alongside the rationale for any categorisation of variables) is described in Table S3 within Supplementary File 1.

"Missing data was observed in six variables, including service user-reported quality of care (1.6%), and ranged from 0.4% - 10.2% of total entries. To assess if missing data in the primary outcome was clustered by facility, we conducted a Chi-square test of independence between the missingness indicator and facility."
and
"Individuals with missing outcome or covariate data were excluded from regression models"

Can the authors please comment on whether missing data can be considered to be missing at random?
Can the authors please consider a sensitivity analysis on their approach to missing

data (applying multiple imputation, for instance)?

Please see our response to comment 12 from Review 1 where we believe we now address this issue.

"Table 2 – Sample and facility characteristics"
Can race or ethnicity please be reported in the study?

We did not collect data on ethnic groups during this study. We sampled facilities from 15 districts and all 3 regions in Malawi which we believe would ensure recruitment of participants across Malawi's different ethnic groups.

VERSION 2 - REVIEW

**Reviewer**	**1**
**Name**	**Vanhamel, Jef**
**Affiliation**	**University of the Witwatersrand Johannesburg, School of Public Health**
**Date**	**10-Jun-2026**
**COI**	

Comments have been successfully addressed by the authors and I have no further concerns. I congratulate the authors on their work.
